# Sequence analysis of the ATP synthase of subunits (*ATP8* and *ATP6*) genes of mitochondrial DNA genome from *Ailuropoda melanoleuca*

**DOI:** 10.1080/23802359.2018.1424574

**Published:** 2018-10-09

**Authors:** Yaodong Hu, Huizhong Pang, Shanshan Ling, Rongping Wei, Yun Zhu, Hemin Zhang, Diyan Li, Desheng Li, Chengdong Wang

**Affiliations:** a Farm Animal Genetic Resources Exploration and Innovation Key Laboratory of Sichuan Province, Sichuan Agricultural University, Chengdu, China;; b China Conservation and Research Center for the Giant Panda, Wolong, China

**Keywords:** *Ailuropoda melanoleuca*, ATP8, ATP6, population genetic, potential phosphorylation site

## Abstract

To explore the effects of the mutations of *ATP6* and *ATP8* genes on energy metabolism and genetic structure, we sequenced the *ATP6* and *ATP8* genes of *Ailuropoda melanoleuca*. Our results showed that *ATP8* is a conserved gene and *ATP6* gene is positively selected during the evolution of the giant panda population with a low genetic diversity. Population expansion was observed in the giant panda group. The T179C mutation on Haplotype7 made the production of a potential phosphorylation site. This non-synonymous mutation may occur at the post-translational modification site that have a potential effect on the function of ATP synthase, related to the maintenance of body temperature of pandas at low metabolic rates.

As the unique mutation of *DUOX2* gene in giant panda (*Ailuropoda melanoleuca*) may explain these low thyroid hormone levels resulting a very low energy metabolism rate (Nie et al. 2015), the question how pandas maintain temperature under low metabolism rate was followed in this study. ATP synthase is core for oxidative phosphorylation, which also affect the normal body temperature (Elston et al. 1998). Proteins encoded by *ATP6* and *ATP8* featured prominently in ATP synthase as an indispensable part of the energy generation, thus we speculate that mutations of those two genes may trigger a series of biological problems. In this study, we aim to investigate the effects of the mutations in *ATP6* and *ATP8* genes on energy metabolism and genetic structure.

Blood samples were collected from 45 giant pandas in Wolong, Sichuan, China. Total genomic DNA was isolated using QIAamp DNA Blood Mini Kit (QIAGEN, Hilden, Germany). Primers for PCRs were designed according to GenBank (accession number: NC_009492.1).

The complete *ATP8* (Genbank accession number: KY978453) and *ATP6* (Genbank accession numbers: KY978454–KY978460) gene nucleotide sequences of giant panda are 204 bp and 681 bp with 49 bp overlap. No mutation observed in *ATP8* indicating it was a conserved gene, this was consistent with Mishmar’s study (Mishmar et al. 2003) in human mtDNAs. *ATP6* gene generated seven haplotypes (Haplotype1-7) based on its seven polymorphic sites (C120T, C166T, T179C, C390T, A418G, G637A, and T664A). Neutral test showed a population expansion in the giant panda group and the *ATP6* gene was positively selected during the evolution. Among the five non-synonymous mutations, the T179C – making the Isoleucine on position 60 of the *ATP6* protein mutanted to Serine – leads to the generation of a potential phosphorylation site on Haplotype7 (Figure 1). Phosphorylation is one of the most important post-translational modification, involved in protein activation or inhibition in different cellular activities (Li et al. 2014). Non-synonymous mutations that occur at the post-translational modification site may have an effect on the associated modification function, resulting a change in protein function (Pan et al. 2014). Phosphorylation mutations in the ATP synthase β subunit affect the conformation of the F1 and decrease the catalytic activity of ATP synthase; The c subunit of F0 is also reported to be phosphorylated (Kane et al. 2010). Combined with the strong positive selection of the *ATP6* gene above, We speculated these five non-synonymous mutations, especially the T179C on *ATP6* gene may cause some altered ATP synthase function, which is likely to bring a change in the process of oxidative phosphorylation. Expression of *ATP6* gene and disorder of oxidative phosphorylation are closely related to reactive oxidative species (ROS) level (Petros et al. 2005; Zhao et al. 2011). Simultaneously, ROS level regulates oxidative phosphorylation uncoupling, and controls basal metabolic heat production through proton leaks. Thus, the T179C on *ATP6* gene was suspected to have relationship with the maintenance of body temperature in pandas at the situation low metabolic rates. Our study gives a possible reason for body temperature maintenance of pandas at low metabolic rates. However, due to the limitations of population in this study, the actual functional site and the specific impact on ATP synthase need further explored.

**Figure 1.**
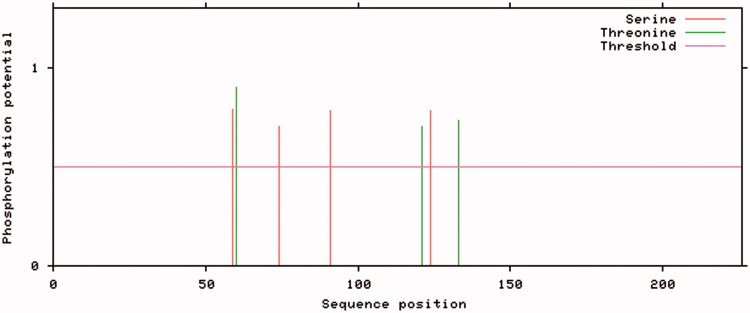
Potential phosphorylation sites of ATP6 on Haplotype7. Four Serine and three Threonine potential phosphorylation sites on Haplotype 7.
